# Isolated IgG4 Autoimmune Cholangitis in the Absence of Pancreatitis

**DOI:** 10.7759/cureus.22754

**Published:** 2022-03-01

**Authors:** Ariana R Tagliaferri, Heemani Ruparel, Gabriel Melki, Yana Cavanagh, Matthew A Grossman

**Affiliations:** 1 Internal Medicine, St. Joseph's Regional Medical Center, Paterson, USA; 2 Medicine, St. Joseph's Regional Medical Center, Paterson, USA; 3 Gastroenterology, St. Joseph's Regional Medical Center, Paterson, USA; 4 Interventional Gastroenterology, St. Joseph's Regional Medical Center, Paterson, USA

**Keywords:** sclerosing cholangitis, jaundice, abdominal pain, autoimmune pancreatitis, autoimmune cholangitis, igg4

## Abstract

The IgG4-related disease is an uncommon immune-mediated condition affecting multiple organ systems concomitantly; however, it is very rare for a patient to have isolated IgG4 autoimmune cholangitis or IgG4-related disease confined to the hepatobiliary system. The majority of cases are associated with pancreatitis and are incidentally discovered in the workup of acute or chronic pancreatitis. When it affects the hepatobiliary system, it develops as inflammatory fibrosclerosing cholangitis, which can mimic other hepatobiliary diseases such as primary sclerosing cholangitis. Herein, we present a case of type 1 IgG4 autoimmune cholangitis in the absence of pancreatitis. Our case is particularly unique because type 1 is the most common type associated with autoimmune pancreatitis; however, our patient had type 1 without any evidence of pancreatic involvement. Additionally, like most cases of isolated IgG4 autoimmune cholangitis, our patient was refractory to standard therapy. This case highlights the clinical significance, rarity and severity of isolated IgG4 autoimmune cholangitis.

## Introduction

Disclosure: This article was previously presented as a meeting abstract at the ACG Annual Meeting in Las Vegas on October 22-27, 2021. The full manuscript below was written by the same authors.

Immunoglobulin G4 (IgG4) related disease (RD) is a multi-organ system inflammatory disorder [1-3]. IgG4 RD has been reported with multi-organ involvement in more than 60% of patients diagnosed with IgG4 RD and is most commonly seen with autoimmune pancreatitis (AIP) [3]. Other organ involvement can include the kidneys, salivary gland, orbit, breast, pericardium, aorta, skin, lungs, prostate or meninges [3].

The hepatobiliary manifestation is a type of fibroinflammatory sclerosis cholangitis, characterized by lesions and/or strictures [1]. IgG4 autoimmune cholangitis (AC) typically presents in the fifth or sixth decades of life with a male predominance [1]. In patients with concomitant autoimmune diseases, IgG4 tends to present in females [3]. Because IgG4 affects many organs simultaneously, the true prevalence is not known. However, a study in Japan estimated the annual prevalence of IgG4-RD in AIP patients as 4.6/100,000 of the population in 2011, with 10.3% of patients found to have porta hepatis involvement and 23.5% having intrahepatic disease [1]. Of all cases, only 8% of IgG4 RD accounts for isolated IgG4 AC [1-4]. A history of allergy, atopy and other autoimmune diseases have been described in isolated IgG4-AC [3].

Clinical presentation is variable depending on the organs involved; however, IgG4-AC may present with obstructive jaundice, weight loss and/or abdominal pain [1-3]. It may be difficult to differentiate IgG4-AC from other causes of biliary obstruction, such as primary sclerosing cholangitis, pancreatic adenocarcinoma or cholangiocarcinoma [1]. To help differentiate from primary sclerosing cholangitis, IgG4-AC tends to involve the distal bile duct, manifests with sudden and acute jaundice rather than insidious onset, and there is a predominance of lymphocytes present on histology compared to primary sclerosing cholangitis [2]. If IgG4-AC is diagnosed with concomitant AIP, patients may present with exocrine or endocrine pancreatic insufficiency [2]. Patients are diagnosed initially through laboratory investigations with findings of abnormal liver enzymes, elevated inflammatory markers, hypergammaglobulinemia and elevated antinuclear antibodies, though none of these laboratory tests are specific [1,3]. The gold standard is through biopsy-proven investigations, in which the three histological hallmarks of IgG4 RD are (1) IgG4 lymphoplasmacytic tissue infiltrates, (2) storiform fibrosis and (3) obliterative phlebitis [3]. It is important to note that endoscopic retrograde cholangiopancreatography (ERCP) is a poor discriminator to differentiate IgG4-AC from other causes of biliary obstruction and thus further imaging and biopsies should be obtained to aid in diagnosis and reveal evidence of other organ involvement [1,2]. Liver biopsy will reveal intra-hepatic involvement in up to 26% of cases and sampling of the biliary fluid is recommended [1]. The HISTORt (histology, imaging, serology, other organ involvement) Diagnostic Criteria is a culmination of these findings [1]. 

Isolated IgG4-AC, as a single organ involvement, is very rare. Herein, we present a case of isolated IgG4-AC.

## Case presentation

A 68-year-old Hispanic male with a past medical history of type 2 diabetes mellitus, hypertension, hyperlipidemia, ankylosing spondylitis, and benign prostatic hyperplasia presented to the Emergency Department (ED) with complaints of abdominal pain for three days prior to presentation. The pain was located in the right upper quadrant (RUQ) and was associated with fatigue, poor appetite, nausea and an objective fever of 103̊ F, lasting four days. On arrival, the patient was afebrile with a blood pressure of 140/76 mmHg, heart rate of 95 beats per minute, respiratory rate of 16, saturating 99% on room air. On the physical exam, the patient was awake, alert, and in mild acute distress. His skin, tongue and eyes were jaundiced. On the abdominal exam, there was epigastric and RUQ tenderness upon superficial and deep palpation without rigidity or guarding, no evidence of organomegaly and normoactive bowel sounds [4].

Initial labs were remarkable for leukocytosis (15.2x10^3^/mm^3^) with left shift, normocytic anemia (hemoglobin 9.7 g/dL) and mild hyponatremia (132 mEq/L), although once corrected for glucose was normal. Liver function tests revealed elevated ALP (556 unit/L), AST (136 unit/L), ALT (331 unit/L) and unconjugated hyperbilirubinemia (total bilirubin 2.1 mg/dL). Lipase levels were within normal limits (22 units/L). Given the history of subjective fevers, RUQ abdominal pain with obstructive jaundice and mixed cholestatic/hepatic liver function abnormalities, the patient was subsequently admitted for acute cholangitis. Blood cultures were negative, and the patient was started on intravenous Piperacillin-sulbactam 3.375 milligrams, every 8 hours for ascending cholangitis. Computerized tomography (CT) of the abdomen/pelvis showed no acute abnormalities, including no evidence of pancreatitis. Magnetic resonance cholangiopancreatography (MRCP) revealed normal common bile duct, trace intrahepatic ductal dilatation and attenuation of the ducts near the confluence of the common hepatic duct with concerns for an underlying mass [4].

**Figure 1:**
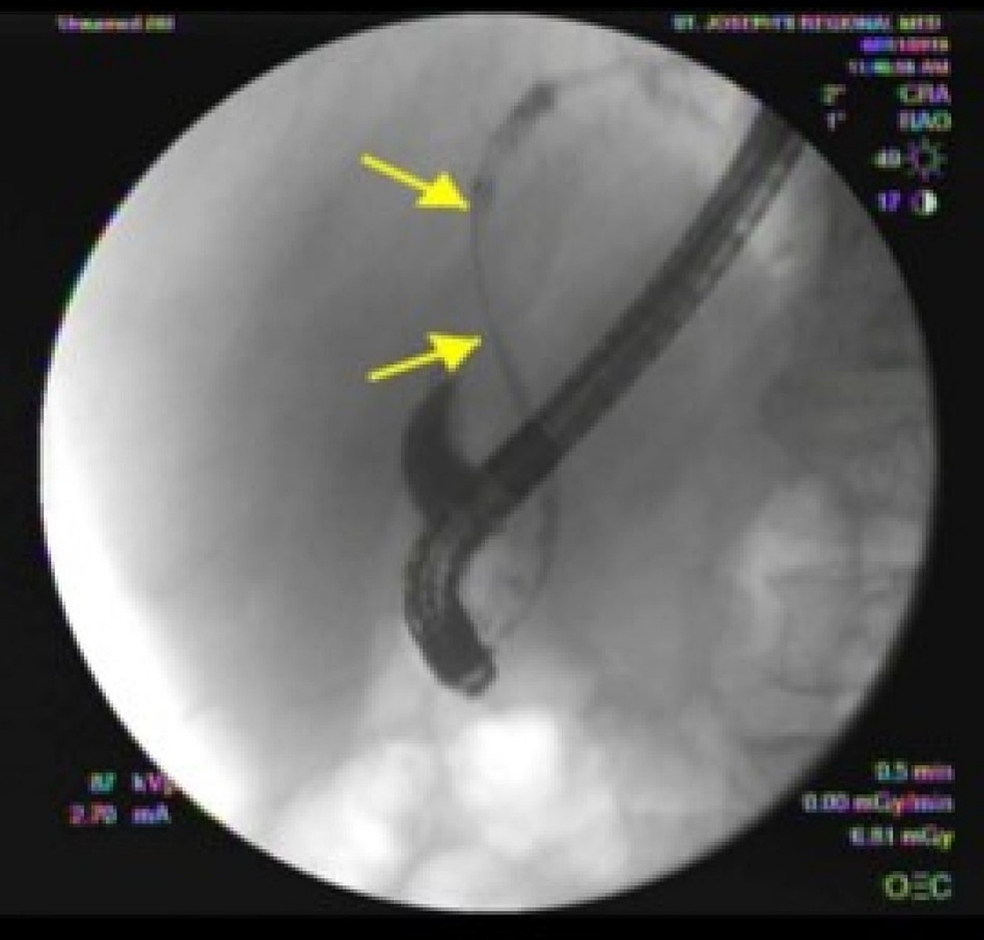
Endoscopic retrograde cholangiography on first admission demonstrating extensive stenosis. The common hepatic duct contained single severe stenosis extending from the level of the bifurcation to the level of the cystic duct take-off (yellow arrows indicate stenosis).

ERCP showed biliary strictures of the common hepatic duct at the level of bifurcation, and the ventral pancreatic duct and common bile ducts were cannulated using plastic stents (Figure 1) [4].

**Figure 2:**
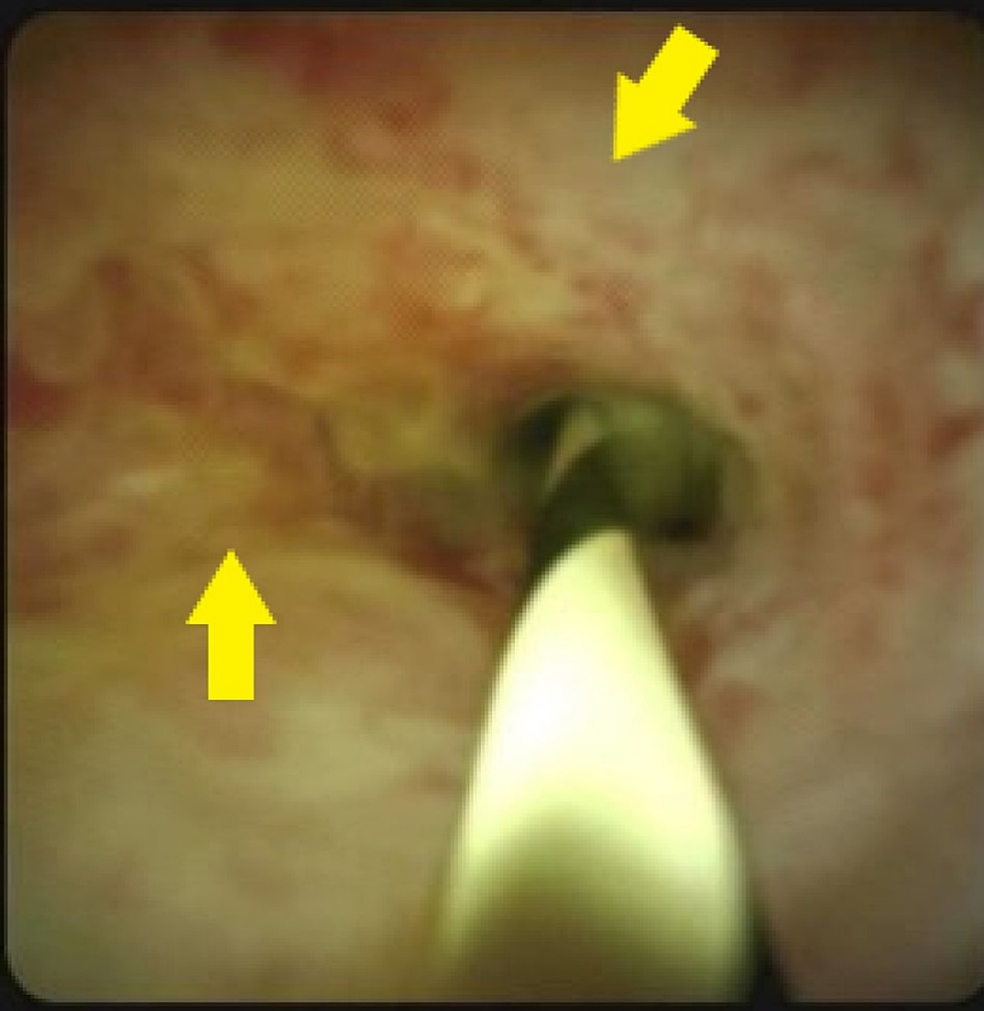
Endoscopic retrograde cholangiography on second admission demonstrating abnormal epithelium of the bile duct hilum. Biopsy from the bile duct hilum showed inflamed and reactive biliary epithelium with rare, atypical cells. Yellow arrows indicate erythema, nodularity and scarring.

The patient was discharged, however, was re-admitted for persistent abdominal pain and further diagnostic workup. Upon readmission, the patient was started again on intravenous Piperacillin-sulbactam and an IgG4 antibody test revealed a level of 400 (abnormal >2.8g/L). At this time, septic workup was again negative, and the patient was subsequently treated with oral Methylprednisolone 40 milligrams daily per standard guidelines. Antibiotics were discontinued. To further aid in diagnosis and evaluation for the extent of involvement, the patient underwent a liver biopsy, which showed extensive portal acute and chronic inflammation with ductular proliferation. The portal inflammation was composed mostly of lymphocytes with scattered neutrophils, focal scattered plasma cells and a few eosinophils. The bile ducts showed focal injuries with ductular proliferation. Prominent interface hepatitis and scattered lobular inflammatory infiltrate with hepatocyte damage were also noted. Biopsy from the bile duct hilum showed inflamed and reactive biliary epithelium with rare, atypical cells (Figure 2).

**Figure 3:**
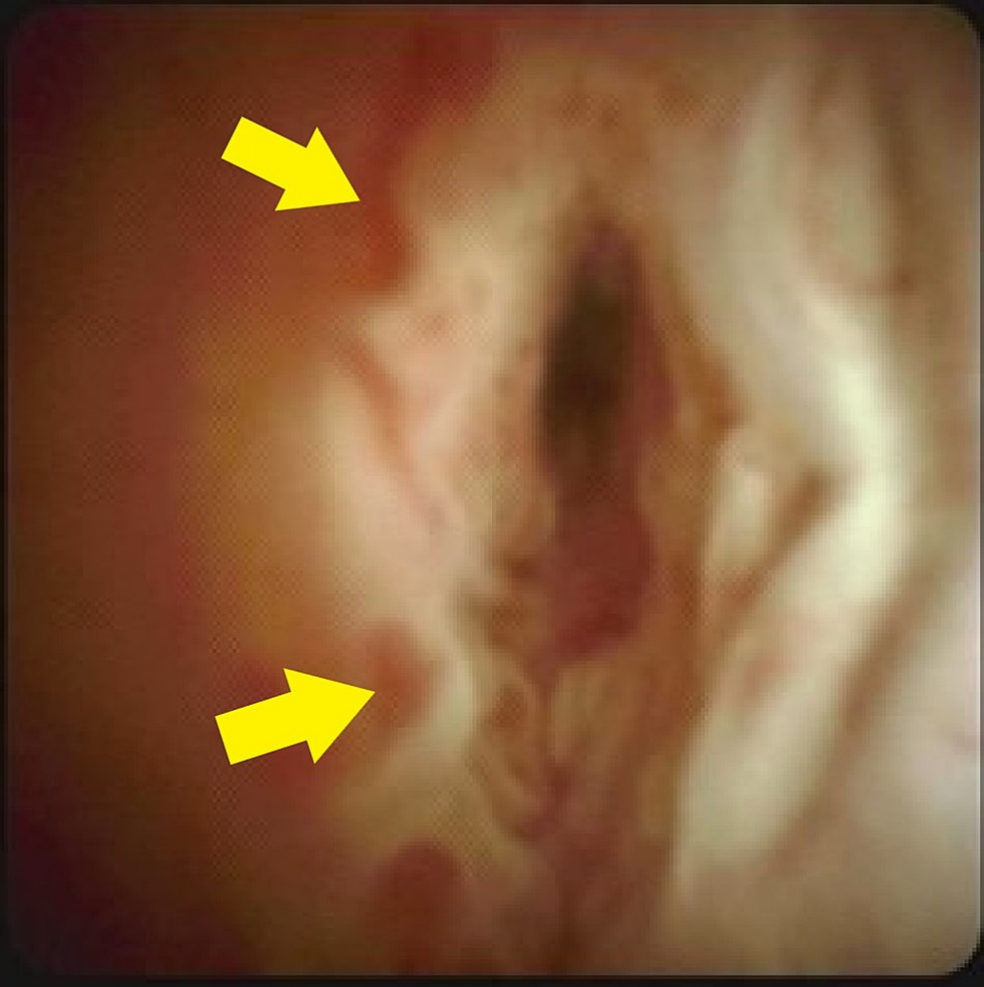
Endoscopic retrograde cholangiography on second admission demonstrating abnormal epithelium of the common hepatic duct. Biopsy of the common hepatic duct showed attenuated biliary epithelium with active inflammation and no malignancy. Yellow arrows indicate the villiform texture of the abnormal epithelium.

Biopsy of the common hepatic duct showed attenuated biliary epithelium with active inflammation and no malignancy (Figure 3).

These findings were compatible with IgG4-related AC, in the absence of pancreatitis [4]. The patient demonstrated significant clinical and laboratory improvement with steroid treatment, and he was subsequently discharged on prednisone at 45 milligrams per day for four weeks. On clinical follow-up, the patient was no longer symptomatic, and his bilirubin levels normalized; however, at his four-month follow-up appointment, liver function enzymes and bilirubin were noted to be elevated again. He was started on rituximab infusions and referred to Rheumatology. Over the next two years, the patient continued to have symptomatic and recurrent obstruction of the intrahepatic ducts and common bile ducts, requiring numerous ERCPs with stenting. He was maintained on Rituximab infusions and steroids without improvement [4].

## Discussion

IgG4 RD most commonly affects the pancreatic and hepatobiliary system and was first identified as a multi-system disease in 2003 in patients with AIP [3]. IgG4 RD occurs in up to 88% of type-1 AIP [3]. Although data are limited on isolated IgG4-AC, it appears as though IgG4-AC accounts for only 8% of Western cohorts [1,4]. If the clinical presentation and histology are similar in both type 1-AIP and IgG4-AC, why do some individuals have a predilection for multi-organ involvement and a rare few with isolated disease?

The pathogenesis of IgG4-AC may involve certain HLA haplotypes overlapping with HLA haplotypes also seen in type-1 AIP, compared to healthy individuals [3]. Additionally, it is thought that IgG4 is up-regulated in response to antigens and pro-inflammatory cytokines, which may explain why you see IgG4 RD in type-1 AIP, or other organ involvement [3]. Conversely, there may be other mechanisms to achieve an isolated IgG4 response in a single organ; however, this process remains unclear [2,3].

Regardless of organ involvement, all laboratory testing remains the same: serum IgG4, and often IgE levels are elevated in more than 50% of patients but are not considered diagnostic [1-3]. Similarly, all organs affected by IgG4 RD have the same histopathological findings, however there are four types of IgG4-AC classifications [1]. Type 1 is most commonly associated with AIP, defined by a low bile duct stricture and/or fibrosis within the head of the pancreas [1]. Type 2 is characterized by a diffuse intrahepatic cholangiopathy and lower common bile duct stricture [1]. Type 3 is defined by a hilar and lower common bile duct stricture, and type 4 is characterized by a hilar stricture alone [1]. Our patient was diagnosed with type 1 classification, because he had filling defects in the pancreatic duct and common hepatic duct. This makes his case more interesting, as he was found to have type 1 isolated IgG4-AC (common hepatic ductal involvement) without pancreatic involvement.

When isolated IgG4-AC is diagnosed, urgent treatment is warranted to prevent permanent fibrosis, infections, venous thrombosis or even death [1,3]. The optimal treatment for IgG4 RD is not well defined, however current recommendations include steroid therapy for four weeks, followed by a taper [1-3]. This treatment has mainly been used in patients with IgG4-AIP with good results [2]. IgG4-AC types 2-4 are more difficult to treat, and other treatment options include azathioprine, methotrexate, or tacrolimus which have been studied in IgG4-AC exclusively [1]. Cyclophosphamide and mycophenolate have been shown to be effective in multi-system IgG4 RD [1]. Our patient was refractory to steroid treatment, despite having type 1 IgG4-AC and required initiation of rituximab. This highlights the progressiveness in isolated IgG4 disease and can be implied that the delay in this patient’s diagnosis led to chronic fibrosis and recurrent stenosis within the hepatobiliary tract as he required repeated cannulation despite being on steroids and rituximab.

## Conclusions

IgG4 causes infiltration of T cells, leading to progressive fibrosis and organ damage affecting any organ system; however, it most commonly affects the pancreas and biliary tract concurrently due to the hepatopancreatic lymphatic and hematogenous drainage. In the absence of elevated serum IgG4 levels or pancreatic features, one must remember the differential of isolated IgG4-AC. Early recognition of IgG4-AC is crucial as its presentation can mimic pancreatic cancer, hepatobiliary malignancies, PSC or other infectious causes of acute cholangitis. Additionally, there are four types of isolated IgG4 AC, which warrant different treatment options and carry a worse prognosis than when associated with pancreatitis. Thus, an accurate and timely diagnosis is imperative.
